# Transgenerational Transmission of Non-communicable Diseases: How to Break the Vicious Cycle?

**DOI:** 10.7759/cureus.18754

**Published:** 2021-10-13

**Authors:** Stephen C Bronson, Veeraswamy Seshiah

**Affiliations:** 1 Institute of Diabetology, Stanley Medical College and Hospital, Chennai, IND; 2 Internal Medicine/Diabetology, The Tamil Nadu Dr. M.G. Ramachandran Medical University, Chennai, IND

**Keywords:** pregnancy, diabetes, non-communicable disease, vicious cycle, in-utero programming, transgenerational transmission

## Abstract

Non-communicable diseases (NCDs) like diabetes, obesity, hypertension, and cardiovascular diseases are major causes of morbidity and mortality all over the world. In recent decades, NCDs are sweeping steadily across the globe much like a silent yet devastating pandemic. Among other factors, the rising trend in diabetes and related NCDs is also linked to hyperglycemia in pregnancy (HIP). Maternal hyperglycemia acts as an in-utero insult to the developing fetus making the offspring prone to develop NCDs in adulthood. Resistance to the hormones insulin and leptin in the offspring affects the metabolic milieu predisposing the individual to obesity and diabetes. Epigenetic processes like DNA methylation, genomic imprinting, and histone modifications are likely to be impacted in an in-utero environment influenced by maternal hyperglycemia. HIP affects not only the health of the mother and her offspring but also sets up adverse intra-uterine programming that leads to a vicious cycle of transgenerational transmission of obesity, insulin resistance, diabetes, and other related NCDS to future generations. The need to break this vicious cycle is much essential now, more than ever before. Children, adolescents, and young adults should be encouraged to maintain a healthy weight and adopt a healthy lifestyle. Preconception counseling should begin early for women with diabetes, with continued guidance throughout their reproductive years. This article highlights how targeting pregnancy-related diabetes to break the chain of transgenerational transmission of NCDs would be an effective action to bring down the increasing burden of NCDs.

## Editorial

Introduction

The ongoing coronavirus disease 2019 (COVID-19) pandemic has shown almost everyone on the planet as to what an adverse impact a widespread, global public health crisis could exert on the lives of people. Drawing an analogy from this communicable disease pandemic, one would better be able to appreciate the silent "pandemic of non-communicable diseases" (NCDs) - especially diabetes and related conditions like obesity, hypertension, and cardiovascular diseases (CVDs) - that is sweeping across the world, rapidly yet steadily over the past few decades.

This paper highlights how maternal hyperglycemia in the setting of pregnancy leads to in-utero metabolic programming of the fetus, thus setting off a vicious cycle leading to a transgenerational risk for NCDs. Physicians and other care providers for pregnant women with diabetes and prediabetes need to be aware of how comprehensive management of dysglycemia in pregnancy would benefit the generations to come.

Searching for the headwaters

CVDs are the leading cause of mortality around the world. Hypertension, smoking, obesity, diabetes, and dyslipidemia are major modifiable risk factors for CVDs. The International Diabetes Federation (IDF) estimates the global prevalence of diabetes in the 20-79 years age group to be 463 million (9.3%) in 2019. On extrapolating the data to the year 2045, the IDF predicts that about 700 million people will be living with diabetes, with a global prevalence of 10.9%. Furthermore, as of 2019, about 7.5% of the adult population had impaired glucose tolerance, which is a pre-diabetic state [1]. Obesity is recognized nowadays as a worldwide health problem and it contributes directly to cardiovascular risk factors like dyslipidemia, hypertension, diabetes, and sleep disorders.

There is a saying in the ancient Tamil language that "one should not search for the origin of a sage and the headwaters of a river" - because of the difficulty in discovering these. On the contrary, in the case of diabetes and other NCDs, a fervent search for the headwaters is much essential now, more than ever before.

While the rising trend in NCDs is attributed to manifold reasons including an aging population, genetic predisposition, urbanization, and nutritional and lifestyle factors [2], one factor that is all the more important is diabetes that occurs in the setting of pregnancy. "Hyperglycemia in pregnancy" (HIP) includes the following: pure gestational diabetes, overt-diabetes detected during pregnancy, and pregnant women with pre-existing diabetes. In 2019, the global prevalence of HIP was 15.8% of all live births [1]. Women with gestational diabetes are at an increased risk of developing diabetes within three to six years after delivery. Children who are exposed to hyperglycemia in-utero are predisposed to a high risk of developing obesity, insulin resistance (IR), and diabetes in the future [2].

Programming inside the womb

In the 1990s, the British physician-cum-epidemiologist David Barker put forward his now-famous hypothesis of “fetal origins of adult disease (FOAD)”. Barker stated that an individual’s susceptibility to many of the adult-onset diseases had already been programmed while he/she was still a fetus inside the mother’s womb [3].

Mothers with HIP are at an increased risk of delivering a large for gestational age (LGA) or a small for gestational age (SGA) neonate. The severe HIP is associated with vasculopathy and nephropathy, which are known to cause intra-uterine growth restriction (IUGR). However, in most cases, HIP leads to increased delivery of glucose to the fetus causing fetal hyperinsulinemia and increased adiposity [4].

According to the FOAD hypothesis, low birth weight, which is a surrogate marker of fetal undernutrition and IUGR, is linked to decreased beta-cell mass in the pancreas and IR in skeletal muscle, liver, and adipocyte. When the IUGR child, after birth, is exposed to adequacy or surplus of nutrients in the external environment, he/she develops an increased risk of obesity, IR, and diabetes in adulthood [3,4]. Leptin, a hormone secreted by adipocytes, is a satiety factor that acts on the central nervous system causing an increase in energy expenditure and a decrease in food intake. Leptin levels are low at birth in an SGA neonate. However, the SGA neonate undergoes a period of "catch-up" growth later during which adiposity increases and the individual becomes hyperleptinemic. Hyperleptinemia is due to decreased and/or resistant leptin receptors in the hypothalamus and this "leptin resistance" leads to increased appetite and decreased energy expenditure, thereby predisposing the individual to diabetes and other NCDs [4].

Of late, moving beyond IUGR, the FOAD hypothesis is implicated in relation to other intra-uterine insults, like maternal hyperglycemia, that results in an adverse in-utero environment [4]. HIP leads to increased delivery of glucose and other macronutrients to the fetus resulting in an over-production of insulin by the fetus. The fetal hyperinsulinemia, in turn, increases fetal glucose utilization and fat deposition thereby leading to macrosomia. Furthermore, the increased glucose utilization by the fetus results in an increased glucose gradient across the placenta, thereby increasing the flux of glucose to the fetus. The increased glucose flux, in turn, leads to increased insulin secretion by the fetal pancreas [2]. Thus, infants born to HIP mothers tend to have increased adiposity, with increased insulin levels [2,4]. Thus, this adverse "intra-uterine programming" predisposes the child to obesity, IR, and diabetes later on in adult life.

Even those infants of HIP mothers who are neither large nor small for gestational age and appear otherwise normal would have been subjected to in-utero metabolic and hormonal perturbations, which favor abnormal, excessive deposition of adipose tissue and predispose the offspring to future disease [4].

Furthermore, epigenetic modifications in response to overnutrition may affect genes involved in the regulation of energy homeostasis. "Epigenetics" deals with processes that influence gene function without altering the DNA sequence. Post-translational DNA modifications like DNA methylation, genomic imprinting, and chromatin changes involving histones result in differential levels of gene expression. During gestation, the epigenetic processes are susceptible to alterations because the rate of DNA synthesis is high and the DNA methylation pattern is established during this time. Genes for leptin, suppressor of cytokine signaling 3 (SOCS3), and glucose transporters are regulated by DNA methylation and histone modifications. Placental leptin gene DNA methylation is found to be correlated with maternal hyperglycemia. Thus, the epigenome of the offspring is likely to be impacted by the in-utero environment of a woman with obesity and/or diabetes [5].

Eventually, the adult, who was once the offspring of a mother with HIP, may develop IR, pre-diabetes, or diabetes. He/she also becomes prone to develop other related NCDs like hypertension, dyslipidemia, and CVD.

Transgenerational effects and the vicious cycle

**Figure 1:**
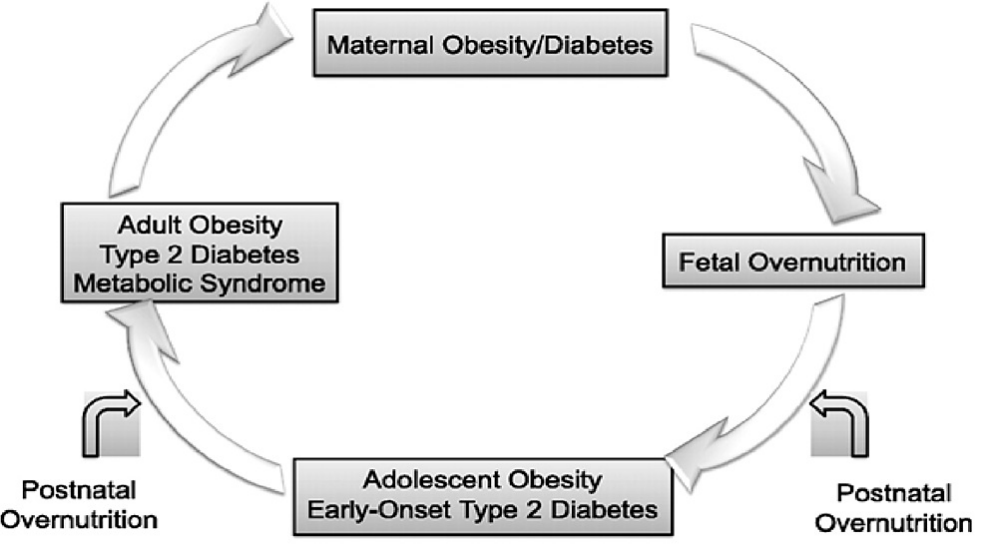
Transgenerational transmission of diabetes – the vicious cycle. From Dabelea and Crume (2011) [5], used under the Creative Commons license - http://creativecommons.org/licenses/by-nc-nd/3.0/.

The claws of HIP extend even beyond the mother and her child and reach future generations. The offspring who was exposed to maternal diabetes/obesity in-utero carries a risk of developing obesity, IR, and diabetes at a younger age [5]. This is beyond the genetic risk carried by the individual to develop diabetes, in a given population [2]. If the offspring were a girl, she would be prone to develop HIP in her childbearing age, thereby facilitating the perpetuation of this vicious cycle (Figure 1) [5].

Obesity begets obesity, diabetes begets diabetes, and the transgenerational vicious cycle goes on. All of this had started at one point - when a woman developed HIP sometime earlier in the lineage.

Pulling up by the roots

Therefore, a major strategic point for checkmating diabetes and other related NCDs lies at the intra-uterine level. To achieve this effect, action should commence well before conception.

The trajectory of development of an individual’s future disease can be impacted very early, even before being conceived in their mother’s womb [4]. Animal experiments with an intake of a diet rich in saturated fat from before conception and continued through weaning led to increased adiposity and hyperinsulinemia in the offspring. Furthermore, human infants of mothers on high glycemic-index diets tend to have higher birth weight and skinfold thickness than those of the mothers on low glycemic-index diets [5]. Therefore, adolescents and young adults, in general, should be encouraged to have an active and healthy lifestyle, maintain an ideal weight, and eat healthy food. As desirable in all pregnancies, preconception counseling needs to be commenced at puberty and continued through reproductive years in women with diabetes. In a woman with pre-existing diabetes, blood sugar values need to be maintained as close to normal levels as possible, prior to conception. She should be encouraged to maintain a glycosylated hemoglobin (HbA1c) level <6.5% (48 mmol/mol) as is safely possible. The first trimester of gestation is a critical period during which organogenesis occurs. If any perturbation occurs at this stage, the damage is likely to persist for a lifetime. If such a perturbation could be thwarted at the earliest, say by achieving good glycemic control in the mother in case of HIP, the risk of future obesity, diabetes, hypertension, and CVD in the offspring could be minimized [2].

Conclusion

The time before and around conception offers a great window of opportunity to optimize metabolic status in all women of reproductive age group and especially in those with pre-existing diabetes. The health of the offspring and further generations depends upon the good metabolic health of the pregnant woman, even from the pre-conception period. Targeting pregnancy-related diabetes and breaking the vicious cycle of transgenerational transmission would be an effective and wholesome action to significantly bring down the expanding burden of diabetes and other NCDs in the world.
